# Giant hydatid cysts in pregnancy: A rare presentation

**DOI:** 10.1590/0037-8682-0500-2020

**Published:** 2021-03-08

**Authors:** Bahar Yılmaz Çankaya, Mustafa Yeşilyurt

**Affiliations:** 1 Atatürk University, Department of Radiology, Erzurum,Turkey.

**FIGURE 1:**
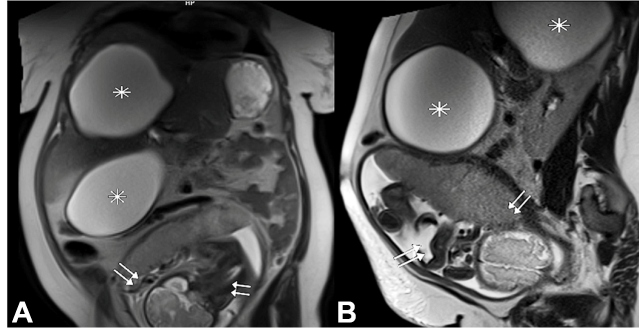
MRI of the abdomen T2-weighted **(A)** coronal and **(B)** sagittal images show hyperintense giant cysts (asterisk) in the liver that compress the uterus and fetus (arrows).

A 23-year-old, 30-week pregnant woman was admitted to our hospital complaining of abdominal pain. Routine laboratory tests were within normal limits. Magnetic resonance imaging (MRI) revealed two unilocular cystic lesions in the VI-VIII and IV segments of the liver of 13 × 14 × 17 cm and 13 × 15 × 16 cm sizes, respectively (Figure 1). The cyst located in the right lobe created pressure on the uterus and fetal sac. The MRI did not detect any other findings that could cause abdominal pain. The patient was followed up for five weeks. Cystectomy and cesarean section were performed under general anesthesia at the 35th week of gestation.

The incidence of hydatid disease in pregnancy is 1 in 20,000-30000^1^. Cyst rupture is one of the complications of hydatid cyst during pregnancy^2^ that can lead to the death of both the mother and fetus due to anaphylaxis. Pregnant women with hydatid cysts should be closely monitored for such complications. Surgery is the preferred method of treatment in cases diagnosed during pregnancy.

The local ethics committee approval was obtained.
